# Heptadecanoic acid and pentadecanoic acid crosstalk with fecal-derived gut microbiota are potential non-invasive biomarkers for chronic atrophic gastritis

**DOI:** 10.3389/fcimb.2022.1064737

**Published:** 2023-01-09

**Authors:** Xiao Gai, Peng Qian, Benqiong Guo, Yixin Zheng, Zhihao Fu, Decai Yang, Chunmei Zhu, Yang Cao, Jingbin Niu, Jianghong Ling, Jin Zhao, Hailian Shi, Guoping Liu

**Affiliations:** ^1^ School of Basic Medical Sciences, Shanghai University of Traditional Chinese Medicine, Shanghai, China; ^2^ School of Computer Science, Fudan University, Shanghai, China; ^3^ Department of Gastroenterology, Shuguang Hospital, Shanghai University of Traditional Chinese Medicine, Shanghai, China; ^4^ Shanghai Key Laboratory of Compound Chinese Medicines, The Ministry of Education (MOE) Key Laboratory for Standardization of Chinese Medicines, The State Administration of Traditional Chinese Medicine (SATCM) Key Laboratory for New Resources & Quality Evaluation of Chinese Medicine, Research Center of Shanghai Traditional Chinese Medicine Standardization, Institute of Chinese Materia Medica, Shanghai University of Traditional Chinese Medicine, Shanghai, China

**Keywords:** chronic atrophic gastritis, gut microbiota, metabonomics, random forest, support vector machine

## Abstract

**Background:**

Chronic atrophic gastritis (CAG), premalignant lesions of gastric cancer (GC), greatly increases the risk of GC. Gastroscopy with tissue biopsy is the most commonly used technology for CAG diagnosis. However, due to the invasive nature, both ordinary gastroscope and painless gastroscope result in a certain degree of injury to the esophagus as well as inducing psychological pressure on patients. In addition, patients need fast for at least half a day and take laxatives.

**Methods:**

In this study, fecal metabolites and microbiota profiles were detected by metabolomics and 16S rRNA V4-V5 region sequencing.

**Results:**

Alteration of fecal metabolites and microbiota profiles was found in CAG patients, compared with healthy volunteers. To identify the most relevant features, 7 fecal metabolites and 4 microbiota were selected by random forest (RF), from A and B sample sets, respectively. Furthermore, we constructed support vector machines (SVM) classifification model using 7 fecal metabolites or 4 gut microbes, or 7 fecal metabolites with 4 gut microbes, respectively, on C sample set. The accuracy of classifification model was 0.714, 0.857, 0.857, respectively, and the AUC was 0.71, 0.88, 0.9, respectively. In C sample set, Spearman’s rank correlation analysis demonstrated heptadecanoic acid and pentadecanoic acid were signifificantly negatively correlated to *Erysipelotrichaceae_UCG-003* and *Haemophilus*, respectively. We constructed SVM classifification model using 2 correlated fecal metabolites and 2 correlated gut microbes on C sample set. The accuracy of classification model was 0.857, and the AUC was 0.88.

**Conclusion:**

Therefore, heptadecanoic acid and pentadecanoic acid, crosstalk with fecal-derived gut microbiota namely Erysipelotrichaceae_UCG-003 and Haemophilus, are potential non-invasive biomarkers for CAG diagnosis.

## Introduction

Chronic atrophic gastritis (CAG) is the final consequence of an inflammatory process which finally results in loss of appropriate mucosal glands (Rodriguez-Castro et al., 2018). CAG is usually considered as premalignant lesions of gastric cancer (GC), and greatly increases the risk of GC (Park and Kim, 2015).

Gastroscopy with tissue biopsy is the most commonly used technology for CAG diagnosis in clinic (Yu et al., 2011; Chooi et al., 2012; Rodriguez-Castro et al., 2018). However, there are several limitations: 1) Gastroscope, including both ordinary gastroscope and painless gastroscope, is invasive, and need at least half a day fasting and even need eat Laxatives, and results in a certain degree of injury to the esophagus (Yu et al., 2011); 2) Ordinary gastroscope often induces nausea and vomiting, which brings psychological pressure to patients; 3) Painless gastroscope needs anesthesia which will be a certain risk, especially for the elderly patients with basic diseases (Schaub and Kern, 2004; Choi et al., 2018; Hao et al., 2020). Therefore, new non-invasive technology for CAG diagnosis in clinic is urgently expected.

Researchers paid more and more attention to dysfunction of metabolites in gastrointestinal diseases especially in GC of rats or patients (Yu et al., 2011; Xu et al., 2017; Zu et al., 2020; Coker et al., 2022; Wang et al., 2022). Metabolites in plasma, such as azelaic acid, glutamate, 2-hydroxybutyrate, urate, creatinine and threonate characterized progressive stages from chronic superficial gastritis (CSG) to GC and might be the potential markers to indicate a risk of GC. (Yu et al., 2011). Many intervention methods in traditional Chinese medicine (TCM) such as Huangqi Jianzhong Tang (Liu et al., 2020), electro-acupuncture and moxibustion (Liu et al., 2017; Xu et al., 2017; He et al., 2018), as well as berberine (Tong et al., 2021) and palmatine (Chen et al., 2020), could modulate metabolites in CAG rats, indicating the potential role of metabolites in pathological process of CAG. However, metabolite profiles for CAG patients has not been well-clarified yet.

The gastrointestinal tract is the site that the gut microbiota interacts with the host. Gut microbiota produces functional molecules like short-chain fatty acids and various metabolites (Morrison and Preston, 2016). Gut microbiota even modulates host metabolism (Morrison and Preston, 2016; Zhang et al., 2018). Gut microbiota disturbance has been also proved to involve in inflammatory bowel diseases which could be recovered by healthy gut microbiota transplantation (Tung et al., 2011; Li et al., 2017). Also, gut microbiota homeostasis benefits the regulation of gastrointestinal function (Cani et al., 2019). Gut microbiota is also proved to involve in the process of CAG in rats (Sgambato et al., 2017). The abundance of bacteria in patients with CAG increased with the reduced secretion of gastric acid and that the changes in intestinal microbiota contribute to the progression from intestinal metaplasia (IM) to gastric cancer (Sharma et al., 1984; Park et al., 2019; Zhang et al., 2019; Zhou et al., 2021). Similar results were also found in CAG rats (Zhou et al., 2021). Therefore, the metabolites-microbiota crosstalk might involve in the pathological process of CAG.

There is a crosstalk between gut microbiota and metabolites (Wang and Zhao, 2018; Jia et al., 2021; Yang and Cong, 2021). However, up to nowadays, there is no research demonstrating the crosstalk between gut microbiota and metabolites in the feces of CAG patients. Therefore, in present study, the microbiota profiles, metabolites profiles and the possible crosstalk between gut microbiota and metabolites in the feces of CAG patients were clarified, and finally the potential non-invasive biomarkers including gut microbiota and metabolites in the feces of CAG patients were also investigated.

## Materials and methods

### Study design and population

**Figure 1:**
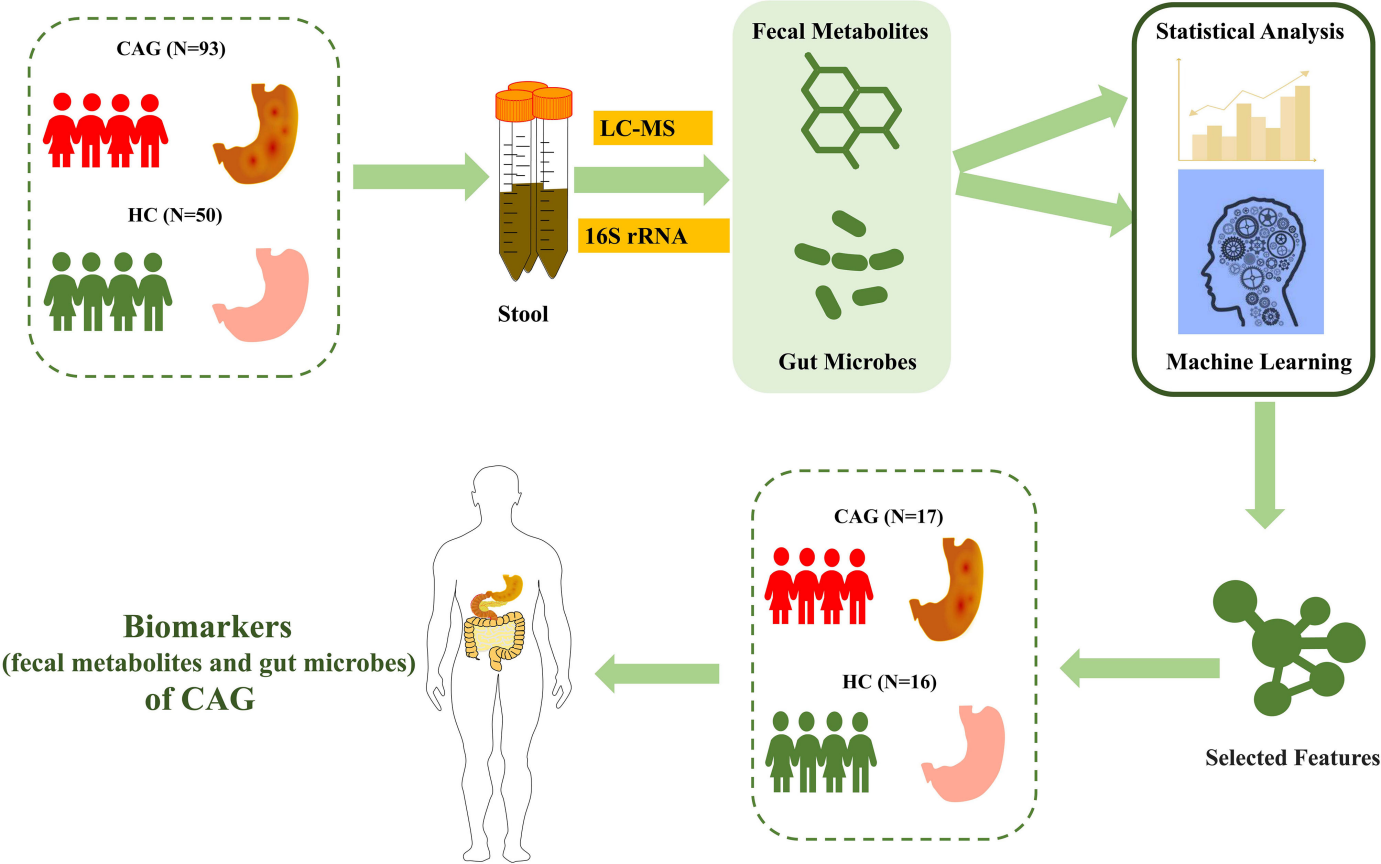
Study design and population. After pathological diagnosis and exclusion process, a total of 176 fecal samples (110 patients with CAG and 66 healthy controls) from Shanghai University of TCM affiliated Shuguang Hospital were prospectively collected. We divided into the discovery phase and validation phase. In the discovery phase, we characterized fecal metabolites among A sample set (30 healthy controls and 48 CAG patients) and gut microbiome among B sample set (20 healthy controls and 45 CAG patients). Furthermore, we identified the markers of gut microbiome and fecal metabolites to construct CAG classifier by random forest model from A sample set and B sample set, respectively. In validation phase, we constructed CAG classification model using 7 fecal metabolites or 4 gut microbes, or 7 fecal metabolites with 4 gut microbes, respectively, on the C sample set (16 healthy controls, 17 CAG patients) to validate diagnosis efficacy.

As shown in **Figure 1**, we consecutively recruited 66 healthy volunteers and 110 CAG patients who received an endoscopic examination in Shanghai University of TCM affiliated Shuguang Hospital, Yueyang Hospital and Longhua Hospital. The fecal metabolites of 78 participants (A sample set) including healthy control group (N=30) and CAG group (N=48) were detected by using ultraperformance liquid chromatography/tandem mass spectrometry (UPLC-MS/MS) system (ACQUITY UPLC-Xevo TQ-S, Waters Corp., Milford, MA, USA). The gut microbes of 65 participants (B sample set) including healthy control group (N=20) and CAG group (N=45) were detected by using 16S rRNA sequencing. In addition, both the profiles of gut microbes and metabolites in feces of 33 participants (C sample set) including healthy control group (N=16) and CAG group (N=17) were detected by using UPLC-MS/MS and 16S rRNA sequencing as a small verification cohort (**Figure 1**). The characteristics of the study population were showed in **Table 1**. There was no significant difference among the CAG group and HC group (A, B and C sample sets) in the gender (p=0.103, P=0.068, P=1.000), mean age (p=0.055, P=0.140, P=0.163), and body mass index (BMI) (p=0.147, P=0.277, P=0.688). The histological assessment was done by the experienced pathologists following clinical guidelines according to “the updated Sydney System” (Dixon et al., 1996). The inclusion criteria were a confirmed diagnosis of CAG according to pathological examination. Patients with gastric polyps, gastric bleeding, gastric tumors, gastrointestinal resection and special gastritis were excluded. This study was approved by the Medical Ethical Committee of Shuguang Hospital (2020-834-41-01). All participants signed the informed consent.

**Table 1:** The characteristics of the study population.

Groups	Gender, male, n (%)	Age, years,median (min-max)	BMI, kg/m^2^, median (min-max)
CAG_a (n=48)	18 (37.50%)	53.67 (35-72)	22.72 (16.33-29.30)
HC_a (n=30)	6 (20.00%)	51.93 (40-72)	22.44 (16.02-28.52)
P values	P=0.103	P=0.055	P=0.147
CAG_b (n=45)	14 (31.11%)	59.20 (38-80)	21.92 (16.33-30.30)
HC_b (n=20)	11 (55.00%)	56.60 (47-80)	22.63 (18.59-27.34)
P values	P=0.068	P=0.140	P=0.277
CAG_c (n=17)	5 (29.41%)	53.47 (40-64)	24.08 (22.49-26.67)
HC_c (n=16)	5 (31.25%)	48.44 (41-56)	24.67 (23.12-27.34)
P values	P=1.000	P=0.163	P=0.688

All clinical information was recorded using the questionnaire made by our study team. Participants were given a fecal sampler and provided detailed illustrated instructions for sample collection. Fecal samples freshly collected from each participant were immediately transported to the laboratory and frozen at -80°C immediately. The biochemical reports of serum were provided by the above hospitals.

### Targeted fecal metabolomics profiling and data processing

All fecal-derived metabolites in this study, were detected by using UPLC-MS/MS with Q300 assay kits for a targeted approach (Metabo-profile Biotechnology, Shanghai, China). All samples were stored at -80°C prior to analysis. The fecal samples were prepared as described previously (Xie et al., 2021). Briefly, the fecal samples were lyophilized, and about 5 mg of each sample was weighed and transferred into a safety lock tube. Homogenization with 25 μl of ultrapure water was followed by extraction with 120 μL of methanol containing internal standards, followed by homogenated for another 3 min and centrifugation at 18 000 g for 20 min. Then the supernatant was transferred to a 96-well plate for derivatization. The following procedures were then performed on an Biomek 4000 workstation (Biomek 4000, Beckman Coulter, Inc., Brea, CA, USA). 20 μL of freshly prepared derivatization reagent was added to each well, and after derivatization at 30°C for 60 min, 330 μL of ice-cold 50% methanol solution was added to dilute the sample, then stored at -20°C for 20 minutes. This was followed by centrifugation at 4 000 g for 30 min at 4°C, and 135 μL of the supernatant from each well was transferred to a new 96-well plate with 10 μL internal standards in each well. All of the standards were obtained from Sigma-Aldrich (St. Louis, MO, USA), Steraloids Inc. (Newport, RI, USA) and TRC Chemicals (Toronto, ON, Canada). A series of standard calibration solutions were diluted for the calibration curve. The calibration curve and the corresponding regression coefficients were obtained by internal standard adjustment. Then, the absolute concentrations of 146 metabolites in fecal samples were detected by UPLC-MS/MS by using Q300 assay kits (Metabo-profile Biotechnology, Shanghai, China).

For mass spectrometer, capillary: 1.5 (ESI+), 2.0 (ESI-) Kv, source temp.: 150°C, desolvation temp.: 550°C, and desolation gas flow: 1 000 L h^-1^. The raw data were deposited into the MetaboLights database (Accession number: MTBLS5990).

For data processing, the raw data files generated by UPLC-MS/MS were processed by using the MassLynx software (v 4.1, Waters Corp., Milford, MA, USA) to perform peak integration, calibration, and quantitation for each metabolite. The calculated absolute concentrations of metabolites were used for univariate analyses and multivariate analyses. Statistical analysis, and pathway analysis were processed on iMAP platform (v1.0; Metabo-Profile, Shanghai, China). A standardized z-score transformation was applied to convert the concentration values to z-scores before analysis in heatmap. Potential biomarkers of differential fecal metabolites were characterized by P<0.05 using student t test or Wilcoxon test based on whether the data were normally distributed between the two groups. Partial least squares-discriminant analysis (PLS-DA) was performed using metaX to discriminate different variables between groups. The logarithmic change (FC) value calculated by comparing the average of the peak area metabolites of both groups. Kyoto Encyclopedia of Genes and Genomes (KEGG) (http://www.genome.jp/kegg/) was used to search and identify important metabolic pathways.

### DNA extraction, 16S rRNA V4-V5 region sequencing and data processing

Microbial community genomic DNA was extracted from fecal samples using the QIAamp DNA Stool Mini Kit according to manufacturer’s instructions. DNA concentration and purity were checked by running the samples on 1.2% agarose gels. Polymerase chain reaction PCR) amplification of 16S rRNA genes was performed by using general bacterial primers (515F 5’-GTGCCAGCMGCCGCGGTAA-3’ and 926R 5’-CCGTCAATTCMTTTGAGTTT-3’). The primers also contained the Illumina 5’overhang adapter sequences for two-step amplicon library building, following manufacturer’s instructions for the overhang sequences. The initial PCR reactions were carried out in 50 μL reaction volumes with 1-2 μL DNA templates, 200 μM dNTPs, 0.2 μM of each primer, 5X reaction buffer 10 μL and 1U Phusion DNA Polymerase (New England Biolabs, USA). PCR conditions consisted of initial denaturation at 94°C for 2 min, followed by 25 cycles of denaturation at 94°C for 30 s, annealing at 56°C for 30 s and extension at 72°C for 30 s, with a final extension of 72°C for 5 min. The second step PCR with dual 8-base barcodes were used for multiplexing. Eight cycle PCR reactions were used to incorporate two unique barcodes to either end of the 16S amplicons. Cycling conditions consisted of one cycle of 94°C for 3 min, followed by eight cycles of 94°C for 30 s, 56°C for 30 s and 72°C for 30 s, followed by a final extension cycle of 72°C for 5 min. Prior to library pooling, the barcoded PCR products were purified by using a DNA gel extraction kit (Axygen, China) and quantified by using the FTC -3000 TM real-time PCR. The libraries were sequenced by 2*300 bp paired-end sequencing on the MiSeq platform using MiSeq v3 Reagent Kit (Illumina) at Tiny Gene Bio-Tech (Shanghai) Co., Ltd. The raw reads were deposited into the NCBI Sequence Read Archive (SRA) database (Accession number: SRP350700).

The raw fastq files were demultiplexed based on the barcode. PE reads for all samples were run through Trimmomatic (version 0.35) to remove low quality base pairs using these parameters (SLIDINGWINDOW: 50:20 MINLEN: 50). Trimmed reads were then cut primer and adaptors by using cutadapt (version:1.16). And then further merged using FLASH program (version 1.2.11) with default parameters. The low quality contigs were removed based on screen. seqs command using the following filtering parameters, maxambig= 0, minlength = 200, maxlength = 485, maxhomop= 8. The 16S sequences were analyzed using a combination of software mothur (version 1.33.3), UPARSE (usearch version v8.1.1756, http://drive5.com/uparse/), and R (version 3.6.3). The demultiplexed reads were clustered at 97% sequence identity into operational taxonomic units (OTUs) by using the UPARSE pipeline (https://drive5.com/usearch/manual8.1/uparse_pipeline.html). The OTU representative sequences were assignment for taxonomy against Silva 128 database with confidence score ≧ 0.7 by the classify.seqs command in mothur.

The data were analyzed on the online platform of Majorbio Cloud Platform (www.majorbio.com) (Ren et al., 2022). For the alpha-diversity analysis, Shannon and Sobs index were calculated. The linear discriminant analysis (LDA) effect size (LEFSe) method was used to analyze significant differences between two groups of bacterial genera on the basis of log10 LDA>2.0. We conducted the Spearman’s rank correlation analysis to predict the correlation between fecal metabolites and gut microbes. We used PICRUSt2 to perform the functional prediction of gut microbiota. First, the OTU abundance was standardized by PICRUSt. Each OTU has its own Greengene ID, then the KEGG Ortholog (KO) information of each OTU was obtained by Greengene ID of each OTU, finally, the abundance of KO was also calculated. According to the KEGG database, PICRUSt can be used to obtain the level information of metabolic pathways, and the abundance table of each level can be obtained respectively.

### Feature selection using the random forest and evaluation using the receiver operator characteristic curves

Feature selection was conducted by using Python version 3.6.12 and machine learning library scikit-learn version 0.23.2. We used random forest (RF) to calculate the importance of 35 fecal metabolites and 27 gut microbes in CAG diagnosis, and sort them in descending order. Then we trained an (support vector machine) SVM classification model circularly with a step size of one, and determine the significant features (biomarkers) of fecal metabolites and gut microbes that make the best performance of classification model on the A and B sample sets. In order to improve the generalization ability and accuracy of the model, we used 5-fold cross-validation and grid search. The discrimination ability of the model was evaluated by using ROC curve, ROC space defines the false positive rate (FPR) as the X axis and the true positive rate (TPR) as the Y axis. a coordinate point (x = FPR, y = TPR) can be calculated by given a binary classification model and a threshold, and all coordinate points of each threshold of a model are drawn in space, which is called the ROC curve of a specific model. The evaluation index is the area under the ROC curve (AUC), The AUC>0.7 indicates that the model has predictive value, the closer the AUC to 1, the better the model performance. In order to verify whether the biomarker of fecal metabolites and gut microbes can well identify the new data set to achieve the purpose of diagnosing CAG, the selected biomarkers were used in the C sample set and established SVM model, The ROC curve was also drawn for evaluation. In the process of selecting significance features based on random forest algorithm and establishing the SVM model, the sample set is divided into 4/5 training set and 1/5 testing set. The code had been deposited in GitHub (https://github.com/fuzh97/SHUTCM-FDU).

### Statistical analysis

The data in text were expressed as mean±standard deviation (m±SD), M (min -max) or M (Q25, Q75). Differences between two groups were analyzed by student *t*-test or Mann-Whitney (U-test) using SPSS25.0, based on whether the data were normally distributed between the two groups. P<0.05 were considered statistically significant.

## Results

### Bile acid, total cholesterol and low-density lipoprotein were higher in serum of CAG patients

As demonstrated in **Table  2**, the levels of bile acid, total cholesterol and low-density lipoprotein in CAG patients were higher than those in healthy volunteers (P< 0.05). However, there was no significant difference in the levels of high-density lipoprotein cholesterol, triglyceride and total bilirubin between healthy and CAG patients.

**Table 2:** Comparison of HDL-C, TG, BA, T-bil, CHOL and LDL-C between CAG_a group and HC_a group [M (Q25, Q75)].

Pathological indexes	CAG_a (n=48)	HC_a (n=30)	P values
High density lipoprotein-cholesterol (HDL-C) (mmol/L)	1.46 (1.41, 1.54)	1.41 (1.25, 1.60)	P=0.550
Triglycerides (TG)(μmol/L)	1.33 (0.90, 1.85)	1.26 (0.83, 1.65)	P=0.590
Bile acid (BA)(mmol/L)	3.10 (1.75, 4.40)	1.85 (1.27, 2.70)	P=0.002**
Total bilirubin (T-bil) (μmol/L)	14.20 (11.63, 16.60)	13.05(9.93, 15.53)	P=0.130
Total cholesterol (CHOL)(mmol/L)	5.67 (5.22, 6.48)	5.10 (4.85, 5.70)	P=0.002**
Low density lipoprotein-cholesterol (LDL-C) (mmol/L)	3.63 (3.41, 4.12)	3.25 (3.00, 3.64)	P=0.004**

Values were expressed as M(Q25, Q75) (n = 5/group, feces; n = 10/group, cecum contents). Data were analyzed by t-test. **P < 0.01 vs. control group.

### Alteration of fecal-derived metabolites profiles in CAG patients

As shown in **Figure S1**, in A sample set, the composition of fecal metabolites in CAG group and HC group was analyzed by metabolomics based on UPLC-MS/MS. A total of 146 metabolites belonging to 16 categories, were identified in fecal samples from CAG_a group and HC_a group, including 31 amino acids, 27 bile acids, 24 fatty acids, 16 organic acids, 10 carbohydrates, 9 SCFAs, 6 benzonic acids, 6 indoles, 5 phenylpropanoic acids, 3 phenols, 2 phenylpropanoids, 2 benzenoids, 2 carnitines, 1 pyridine, 1 DHA and 1 steroids and steroid derivatives.

**Figure 2:**
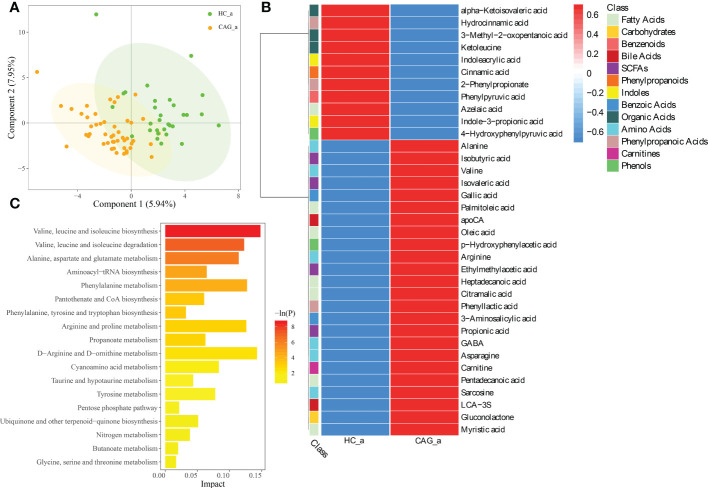
Alteration of fecal-derived metabolites profiles in CAG_a patients. **(A)** PLS-DA indicated the difference of fecal metabolites between CAG_a and HC_a healthy volunteers. **(B)** Alteration of fecal-derived metabolites profiles in CAG_a patients. **(C)** All differential metabolites enriched for metabolic pathways based on KEGG analysis. Values were expressed as mean ± SD (n=30/HC_a, n=48/CAG_a) Data were analyzed by t-test or Wilcoxon test based on whether the data were normally distributed between CAG_a group and HC_a group.

PLS-DA is a versatile algorithm that can be used for predictive and descriptive modelling as well as for discriminative variable selection. In this present study, PLS-DA method was used to reflect the difference of metabolites between HC_a group and CAG_a group, and to investigate the aggregation tendency of the same group and the separation tendency of the different groups. The results demonstrated that there was a separation tendency between HC_a group and CAG_a group. The metabolites in CAG_a group were mainly distributed in the left quadrant, and metabolites in HC_a group were mainly distributed in the right quadrant. The score plots of PLS-DA are shown in **Figure  2A**.

As shown in **Figure  2B** and **Table S1**, there were 35 fecal metabolites in CAG_a group significantly different from that in HC_a group, namely 7 fatty acids (azelaic acid, heptadecanoic acid, palmitoleic acid, pentadecanoic acid, myristic acid, oleic acid, citramalic acid); 6 amino Acids (gamma_aminobutyric acid (GABA), alanine, valine, sarcosine, arginine, asparagine); 4 SCFAs (ethylmethylacetic acid, isobutyric acid, propionic acid, isovaleric acid); 3 phenylpropanoic acids (2-phenylpropionate, phenyllactic acid, hydrocinnamic acid); 3 organic acids (alpha-ketoisovaleric acid, ketoleucine, 3-methyl-2-oxopentanoic acid); 2 benzoic acids (3-aminosalicylic acid, gallic acid); 2 bile acids (lithocholic acid 3 sulfate (LCA-3S), apocholic acid (apoCA)); 2 indoles (indoleacrylic acid, indole-3-propionic acid); 2 phenols (4-hydroxyphenylpyruvic acid, p-hydroxyphenylacetic acid); 1 benzenoids (phenylpyruvic acid); 1 carbohydrates (gluconolactone); 1 carnitines (carnitine); 1 phenylpropanoids (cinnamic acid). Comparing with the fecal metabolites of healthy people in HC_a group, 11 fecal-derived metabolites (cinnamic acid, indoleacrylic acid, 2-phenylpropionate, ketoleucine, azelaic acid, 3-methyl-2-oxopentanoic acid, indole-3-propionic acid, phenylpyruvic acid, 4-hydroxyphenylpyruvic acid, alpha-ketoisovaleric acid, hydrocinnamic acid) were down regulated, and 24 fecal-derived metabolites (alanine, isobutyric acid, valine, isovaleric acid, gallic acid, palmitoleic acid, apoCA, oleic acid, p-hydroxyphenylacetic acid, arginine, ethylmethylacetic acid, heptadecanoic acid, citramalic acid, phenyllactic acid, 3-aminosalicylic acid, propionic acid, GABA, asparagine, carnitine, pentadecanoic acid, sarcosine, LCA-3S, gluconolactone, myristic acid) were up regulated. (P< 0.05).

As shown in **Figure  2C**, KEGG analysis indicated that the differentiated metabolites were mainly focused in Valine, leucine and isoleucine biosynthesis; Valine, leucine and isoleucine degradation; Alanine, aspartate and glutamate metabolism; Aminoacyl-tRNA biosynthesis; Phenylalanine metabolism; Pantothenate and coenzyme A (CoA) biosynthesis; Phenylalanine, tyrosine and tryptophan biosynthesis; Arginine and proline metabolism; Propanoate metabolism; D-Arginine and D-ornithine metabolism; Cyanoamino acid metabolism; Taurine and hypotaurine metabolism; Tyrosine metabolism; Pentose phosphate pathway; Ubiquinone and other terpenoid-quinone biosynthesis; Nitrogen metabolism; Butanoate metabolism; Glycine, serine and threonine metabolism.

**Figure 3:**
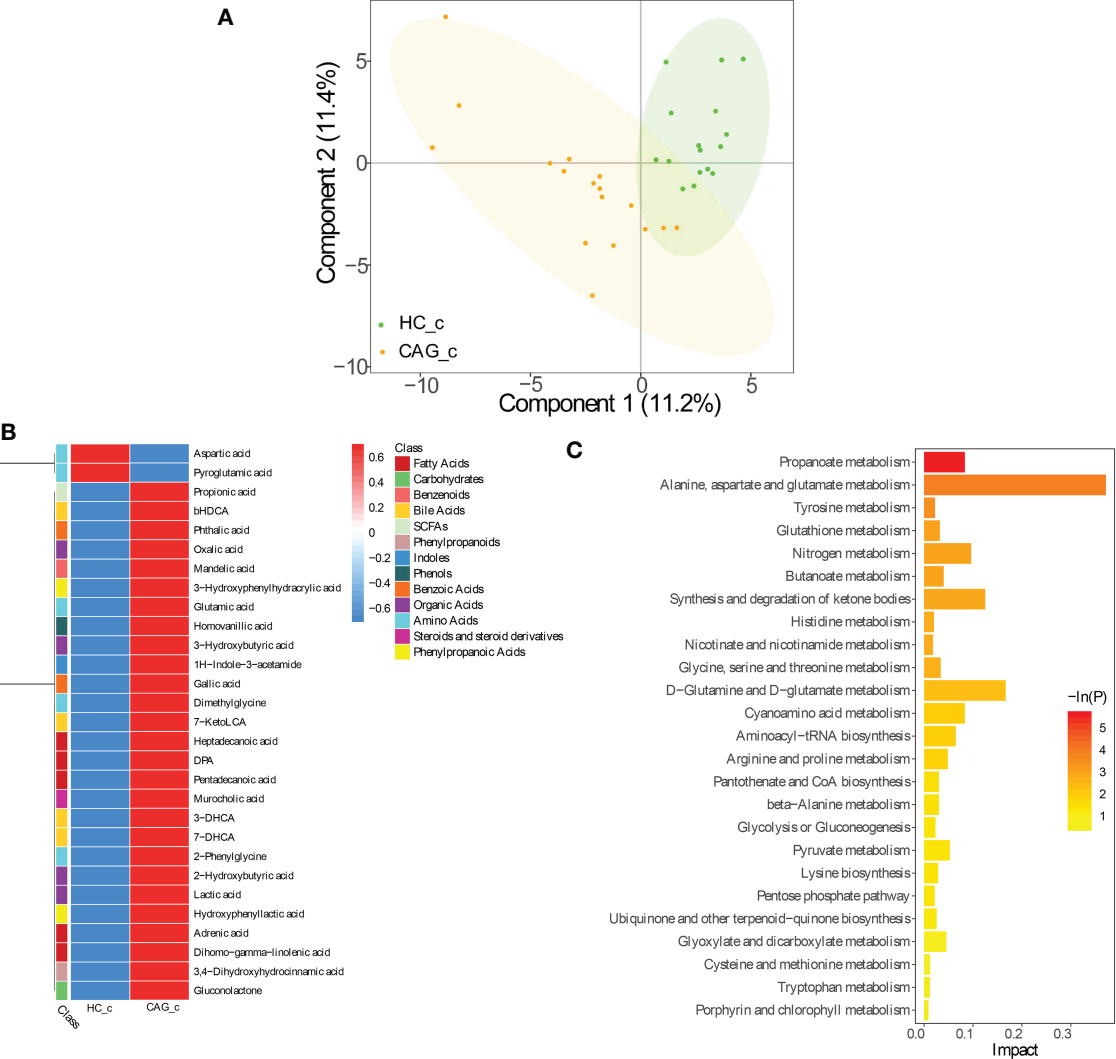
Alteration of fecal-derived metabolites profiles in CAG_c patients. **(A)** PLS-DA indicated the difference of fecal metabolites between CAG_c and HC_c healthy volunteers. **(B)** Alteration of fecal-derived metabolites profiles in CAG_c patients. **(C)**. All differential metabolites enriched for metabolic pathways based on KEGG analysis. Values were expressed as mean ± SD (n=16/HC_c, n = 17/CAG_c). Data were analyzed by t-test or Wilcoxon test based on whether the data were normally distributed between CAG_c group and HC_c group.

As demonstrated in **Figure  3**, PLS-DA assay results demonstrated that there was a separation tendency between HC_c group and CAG_c group. The metabolites in CAG_c group were mainly distributed in the left quadrant, and metabolites in HC_c group were mainly distributed in the right quadrant. The score plots of PLS-DA are shown in **Figure  3A**.

In C samples set, from feces of CAG_c group and HC_c group, there were 29 fecal metabolites belonging to 13 catergories in CAG_c group significantly different from that in HC_c group, namely 5 fatty acids (pentadecanoic acid, dihomo - gamma - linolenic acid, (docosapentaenoic acid) DPA, adrenic acid, heptadecanoic acid); 5 amino acids (glutamic acid, dimethylglycine, 2-phenylglycine, aspartic acid, pyroglutamic acid); 4 organic acids (lactic acid, 3-hydroxybutyric acid, 2-hydroxybutyric acid, oxalic acid); 4 bile acids (7-DHCA (7-dehydrocholic acid), 3-DHCA (3-dehydrocholic acid), bHDCA (beta_hyodeoxycholic acid), 7-KetoLCA (7-ketolithocholic acid)); 2 benzoic acids (gallic acid, phthalic acid); 2 phenylpropanoic acids (hydroxyphenyllactic acid, 3-hydroxyphenylhydracrylic acid); 1 SCFAs (propionic acid); 1 phenylpropanoids (3,4-dihydroxyhydrocinnamic acid); 1 indoles (1H-indole-3-acetamide); 1 phenols (homovanillic acid); 1 benzenoids (mandelic acid); 1 carbohydrates (gluconolactone); 1 steroids and steroid derivatives (murocholic acid). Comparing with the fecal metabolites of healthy people in HC_c group, only 2 fecal-derived metabolites (aspartic acid, pyroglutamic acid) were downregulated, and 27 fecal-derived metabolites (propionic acid, bHDCA, phthalic acid, oxalic acid, mandelic acid, 3-hydroxybutyric acid, glutamic acid, homovanillic acid, 3-hydroxyphenylhydracrylic acid, 1H-indole-3-acetamide, gallic acid, dimethylglycine, 7-ketoLCA, heptadecanoic acid, DPA, pentadecanoic acid, murocholic acid, 3-DHCA, 7-DHCA, 2-phenylglycine, 2-hydroxybutyric acid, lactic acid, hydroxyphenyllactic acid, adrenic acid, dihomo-gamma-linolenic acid, 3,4-dihydroxyhydrocinnamic acid, gluconolactone) were upregulated. (P< 0.05) (**Figure  3B**, **Table S2**).

As shown in **Figure  3C**, KEGG analysis indicated that the differentiated metabolites were mainly focused in Propanoate metabolism; Alanine, aspartate and glutamate metabolism; Tyrosine metabolism; Glutathione metabolism; Nitrogen metabolism; Butanoate metabolism; Synthesis and degradation of ketone bodies; Histidine metabolism; Nicotinate and nicotinamide metabolism; Glycine, serine and threonine metabolism; D-Glutamine and D-Glutamate metabolism; Cyanoamino acid metabolism; Aminoacyl− tRNA biosynthesis; Arginine and proline metabolism; Pantothenate and CoA biosynthesis; beta-Alanine metabolism; Glycolysis or Gluconeogenesis; Pyruvate metabolism; Lysine biosynthesis; Pentose phosphate pathway; Ubiquinone and other terpenoid-quinone biosynthesis; Glyoxylate and dicarboxylate metabolism; Cysteine and methionine metabolism; Tryptophan metabolism; Porphyrin and chlorophyll metabolism.

### Alteration of fecal-derived gut microbiota profiles in CAG patients

**Figure 4:**
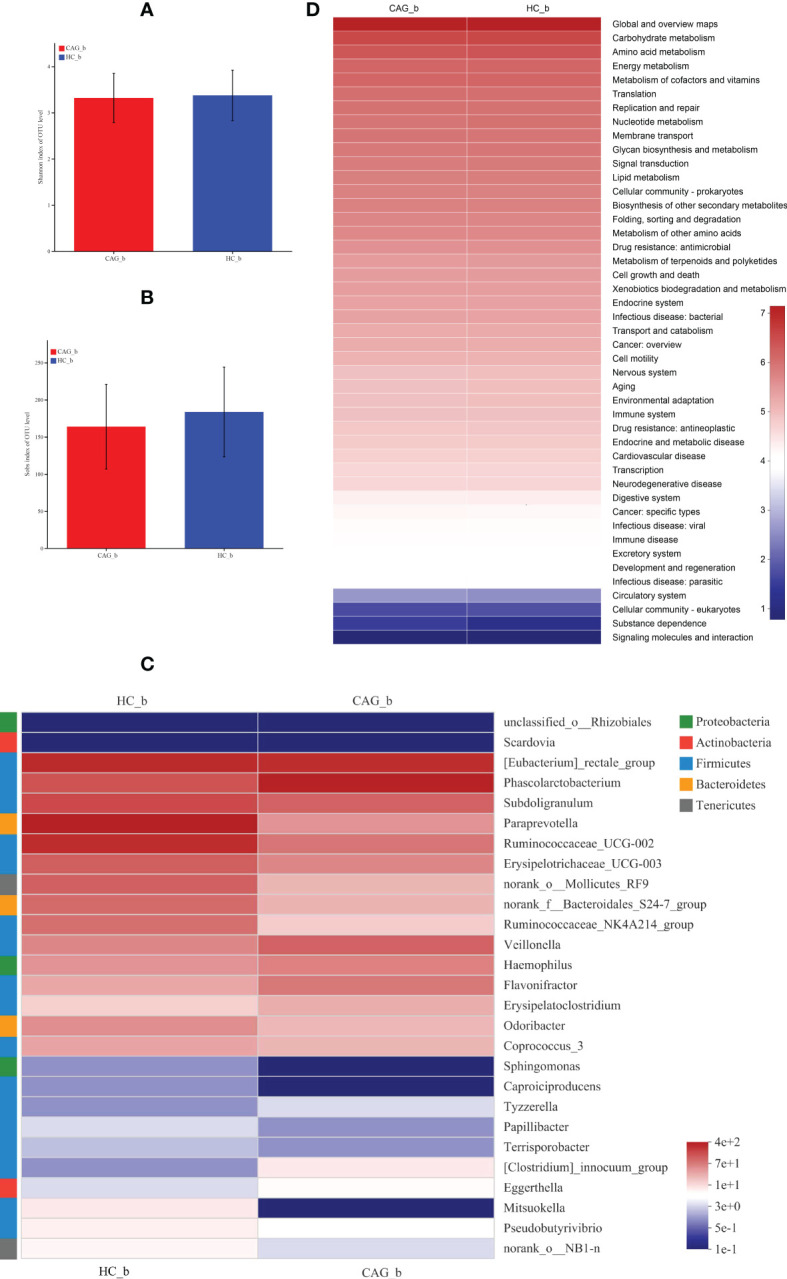
Alteration of fecal-derived gut microbiota profiles in CAG_b patients. **(A)** Shannon index of OTU level indicated there is no difference of α-diversity of gut microbiota in feces of CAG patients, compared with HC_b healthy volunteers. **(B)** Sobs index of OTU level indicated there is no difference of α-diversity of gut microbiota in feces of CAG patients, compared with HC_b healthy volunteers. **(C)** Alteration of fecal-derived gut microbiota profiles in CAG_b patients. **(D)** KEGG analysis indicated the pathways mediated by the differentiated fecal microbiota.

Therefore, to clarify the change of fecal gut microbiota of CAG patients, the diversity and composition of fecal-derived gut microbiota were analyzed by Miseq sequencing. The Sobs index and Shannon index were used to estimate α -diversity. Sequencing of 16S rRNA gene V4-V5 region of gut microbiota showed that there were no difference of Sobs index and Shannon index in feces between HC_b group and CAG_b group (**Figure  4A, B**).

Linear discriminant analysis Effect Size (LEfSe) determines the features (organisms, clades, operational taxonomic units, genes, or functions) most likely to explain differences between classes by coupling standard tests for statistical significance with additional tests encoding biological consistency and effect relevance. (Segata et  al., 2011)

**Figure 5:**
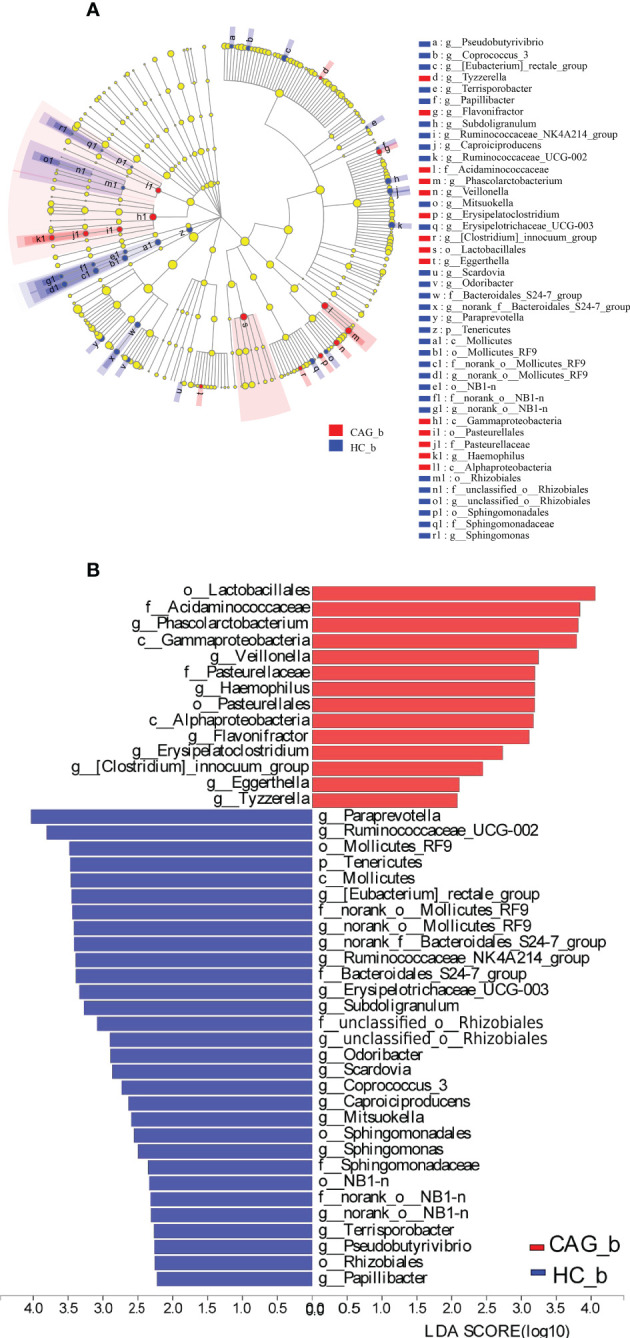
Enriched gut microbiota profiles in feces of CAG_b patients and/or HC_b healthy volunteers. **(A)** Cladogram plot: red nodes indicated significantly enriched bacterial colony with significant impact in CAG patients, and blue nodes indicated significantly enriched bacterial colony with significant impact in HC_b healthy volunteers. Light yellow nodes indicated bacterial colony without significant difference in both CAG patients and HC_b healthy volunteers. **(B)** LDA discriminant analysis histogram: red bar represented the bacterial colony enriched in CAG patients; blue bar represented the bacterial colony enriched in the HC_b healthy volunteers.

Furthermore, in order to further distinguish the difference of intestinal flora between HC_b group and CAG_b group, we used the LEfSe to further analyze the bacterial flora markers with significant difference between the CAG_b group and HC_b group. The level of bacterial taxonomy chosen ranged from phylum to genus, with the threshold value of LDA set at 2, and linear discriminant analysis (LDA) was used to determine the most likely explanation for the difference between the CAG_b group and HC_b group. As demonstrated in **Figure  4C** and **Figure  5**, there were 1 phylum (*Tenericutes*) and 27 genera with significant difference between the CAG_b group and HC_b group (**Figure  5A**).

In 27 genera, 2 genera namely *Eggerthella* and *Scardovia* belonged to the phylum of *Actinobacteria*, 3 genera namely *Paraprevotella, norank_f:Bacteroidales_S24-7_group and Odoribacter* belonged to the phylum of *Bacteroidetes*; 17 genera namely *[Eubacterium]_rectale_group, Phascolarctobacterium, Subdoligranulum, Ruminococcaceae_UCG-002, Erysipelotrichaceae_UCG-003, Ruminococcaceae_NK4A214_group, Veillonella, Flavonifractor, Erysipelatoclostridium, Coprococcus_3, Caproiciproducens, Tyzzerella, Papillibacter, Terrisporobacter, [Clostridium]_innocuum_group, Mitsuokella and Pseudobutyrivibrio* belonged to the phylum of *Firmicutes*, 3 genera namely *Haemophilus, unclassified_o:Rhizobiales and Sphingomonas* belonging to the phylum of *Proteobacteria*, 2 genera namely *norank_o:Mollicutes_RF9 and norank_o:NB1-n* belonged to the phylum of *Tenericutes* (**Figure  4C**). In genus level, we found that 8 genera namely *Phascolarctobacterium*, *Veillonella*, *Haemophilus*, *Flavonifractor*, *Erysipelatoclostridium*, *Clostridium_innocuum_group*, *Eggerthella* and *Tyzzerella* were significantly increased in feces of CAG_b patients, compared with that of HC_b people; however, there were 19 genera namely *Papillibacter*, *Pseudobutyrivibrio*, *Terrisporobacter*, *norank_o_NB1_n*, *Sphingomonas*, *Mitsuokella*, *Caproiciproducens*, *Coprococcus_3*, *Scardovia*, *Odoribacter*, *unclassified_o_Rhizobiales*, *Subdoligranulum*, *Erysipelotrichaceae_UCG_003*, *Ruminococcaceae_NK4A214_group*, *norank_f_Bacteroidales_S24_7_group*, *norank_o_Mollicutes_RF9*, *Eubacterium_rectale_group*, *Ruminococcaceae_UCG_002*, *Paraprevotella* were significantly decreased in feces of CAG_b patients (**Figure  5B**).

Furthermore, as shown in **Figure  4D**, we further analyzed the function of fecal-derived gut microbiota in CAG_b patients by PICRUSt2 analysis, the results indicated that gut microbiota in CAG_b patients mainly involved in carbohydrate metabolism, amino acid metabolism, energy metabolism, metabolism of cofactors and vitamins, translation, replication and repair, nucleotide metabolism, membrane transport, glycan biosynthesis and metabolism, signal transduction, lipid metabolism, cellular community-prokaryotes, biosynthesis of other secondary metabolites, folding, sorting and degradation, metabolism of other amino acids, drug resistance: antimicrobial, metabolism of terpenoids and polyketides, cell growth and death, etc.

**Figure 6:**
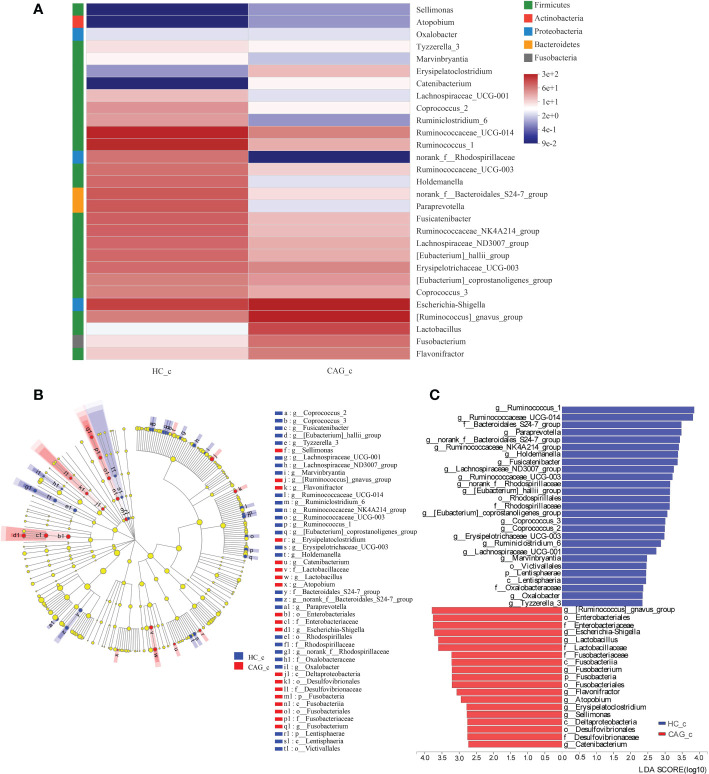
Alteration of fecal-derived gut microbiota profiles in CAG_c patients. **(A)** Alteration of fecal-derived gut microbiota profiles in CAG_c patients. **(B)** Cladogram plot: red nodes indicated significantly enriched bacterial colony with significant impact in CAG_c patients, and blue nodes indicated significantly enriched bacterial colony with significant impact in healthy volunteers (HC_c). Light yellow nodes indicated bacterial colony without significant difference in both CAG patients and healthy volunteers (HC_c). **(C)** LDA discriminant analysis histogram: red bar represented the bacterial colony enriched in CAG_c patients; blue bar represented the bacterial colony enriched in the healthy volunteers (HC_c).

Similar results were obtained in the feces of CAG_c and HC_c samples. In order to further distinguish the difference of intestinal flora between HC_c group and CAG_c group, LEfSe software was used to further analyze the bacterial flora markers with significant difference between the CAG_c group and HC_c group. The level of bacterial taxonomy chosen ranged from phylum to genus, with the threshold value of LDA set at 2, and linear discriminant analysis was used to determine the most likely explanation for the difference between the CAG_c group and HC_c group (**Figure  6**). The results showed that there were 2 phylum (*Fusobacteria*, *Lentisphaerae*) and 29 genera with significant difference between the the CAG_c group and HC_c group (**Figure  6B**). In 29 genera, 1 genus of *Atopobium* belonged to the phylum of *Actinobacteria*, 2 genera namely *Paraprevotella*, *norank_f_ Bacteroidales_ S24-7_group* belonged to the phylum of *Bacteroidetes*, 1 genera of *Fusobacterium* belonged to the phylum of *Fusobacteria*; 3 genera namely *Escherichia-Shigella*, *Oxalobacter*, *norank_f_Rhodospirillaceae* belonged to the phylum of *Proteobacteria*; 22 genera namely *Sellimonas*, *Tyzzerella_3*, *Marvinbryantia*, *Erysipelatoclostridium*, *Catenibacterium*, *Lachnospiraceae_UCG-001*, *Coprococcus_2*, *Ruminiclostridium_6*, *Ruminococcaceae_UCG-014*, *Ruminococcus_1*, *Ruminococcaceae_UCG-003*, *Holdemanella*, *Fusicatenibacter*, *Ruminococcaceae_NK4A214_group*, *Lachnospiraceae_ND3007_group*, *[Eubacterium]_hallii_group*, *Erysipelotrichaceae_UCG-003*, *[Eubacterium]_coprostanoligenes_group, Coprococcus_3*, *[Ruminococcus]_gnavus_group*, *Lactobacillus*, *Flavonifractor* belonged to the phylum of *Firmicutes* (**Figure  6A**). There were 9 genera including *Catenibacterium, Sellimonas, Erysipelatoclostridium, Atopobium, Flavonifractor, Fusobacterium, Lactobacillus, Escherichia-Shigella, [Ruminococcus]_gnavus_group* were significantly enriched in the feces of CAG_c samples; and 20 genera namely *Tyzzerella_3, Oxalobacter, Marvinbryantia, Lachnospiraceae_UCG-001, Ruminiclostridium_6, Erysipelotrichaceae_UCG-003, Coprococcus_2, Coprococcus_3, [Eubacterium]_coprostanoligenes_group, [Eubacterium]_hallii_group, norank_f:Rhodospirillaceae, Ruminococcaceae_UCG-003, Lachnospiraceae_ND3007_group, Fusicatenibacter, Holdemanella, Ruminococcaceae_NK4A214_group, norank_f_Bacteroidales _S24-7_group, Paraprevotella, Ruminococcaceae_UCG-014, Ruminococcus_1* were significantly enriched in the feces of HC_c samples (**Figure  6C**).

### Feature selection using the RF and evaluation using the ROC curves

#### Feature selection of 35 fecal metabolites on the A sample set

**Figure 7:**
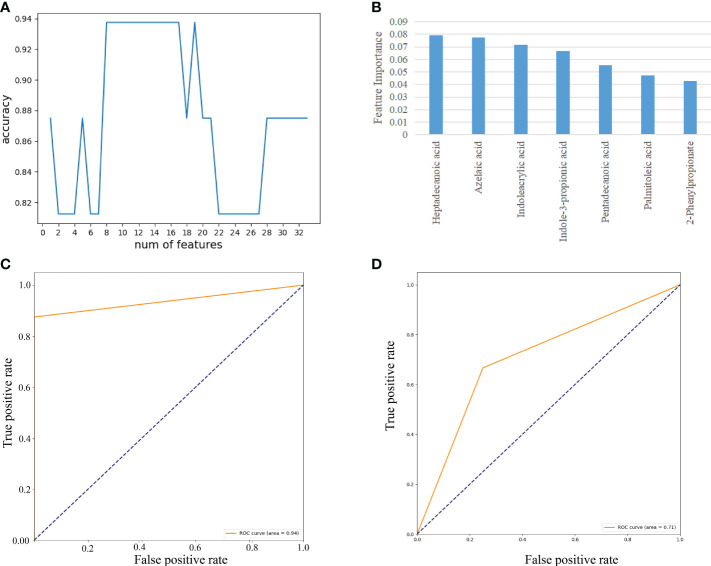
Feature selection of fecal metabolites by using the random forest (RF) and evaluation using the ROC curves. **(A)** The accuracy changes of classification model on the A sample set. **(B)** The importance in descending order (7 fecal metabolites). **(C)** Evaluation using ROC curve on the A sample set. **(D)** Evaluation using ROC curve on the C sample set.

As demonstrated in **Figure  7**, we used RF to calculate the importance of 35 fecal metabolites and trained an SVM classification model on the A sample set. We determined the biomarkers of fecal metabolites according to the best accuracy of classification model (details are shown in the “ Materials and methods” section). When the features were 7 fecal metabolites, the best accuracy of classification was 0.938 (**Figure  7A**). The importance of 7 fecal metabolites in descending order was heptadecanoic acid (0.079), azelaic acid (0.077), indoleacrylic acid (0.071), indole-3-propionic acid (0.067), pentadecanoic acid (0.055), palmitoleic acid (0.047), 2-phenylpropionate (0.043) (**Figure  7B**). Then ROC curves were used to evaluate the classification ability of the model. The results have shown that 7 fecal metabolites could distinguish CAG patients from healthy controls, as indicated by the AUC, which had a value up to 0.94 on the A set (**Figure  7C**). Moreover, we constructed SVM classification model using 7 fecal metabolites on the C sample set. The accuracy of classification model was 0.714. The AUC was 0.71 (**Figure  7D**).

#### Feature selection of 27 gut microbes on the B sample set

**Figure 8:**
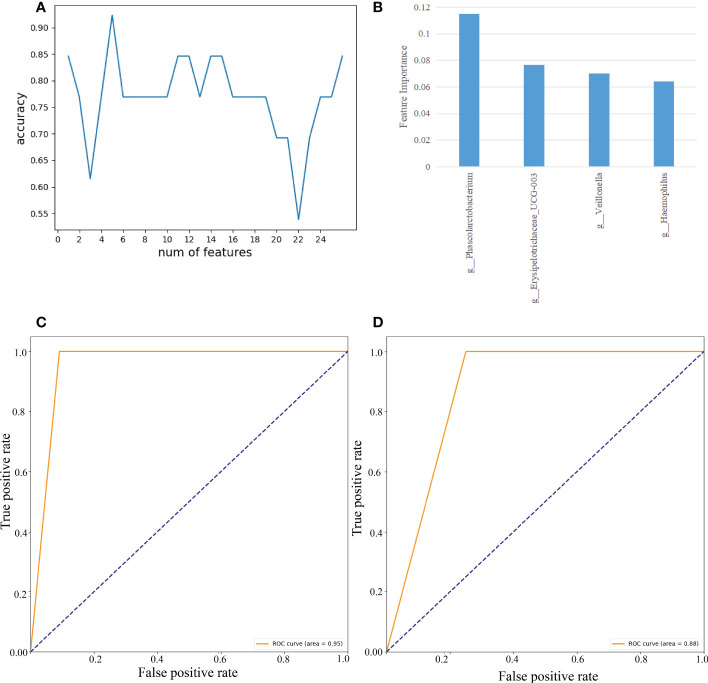
Feature selection of fecal gut microbes on the B sample set by using the random forest (RF) and evaluation using the ROC curves. **(A)** The accuracy changes of classification model on the B sample set. **(B)** The importance in descending order(4 gut microbes). **(C)** Evaluation using ROC curve on the A sample set. **(D)** Evaluation using ROC curve on the C sample set.

As demonstrated in **Figure  8**, we used RF to calculate the importance of 27 gut microbes and trained a SVM classification model on the B sample set. We determined the biomarkers of gut microbes according to the best accuracy of classification model (details are shown in the “ Materials and methods” section). When the features were 4 gut microbes, the best accuracy of classification was 0.923 (**Figure  8A**). The importance of 4 gut microbes in descending order was *g:Phascolarctobacterium* (0.115), *g:Erysipelotrichaceae_UCG-003*(0.077), *g:Veillonella*(0.070), *g:Haemophilus*(0.064)(**Figure  8B**). Then ROC curves were used to evaluate the classification ability of the model. The results have shown that 4 gut microbes could distinguish CAG patients from healthy controls, which had a value up to 0.95 on the A set (**Figure  8C**). Moreover, we constructed SVM classification model using 4 gut microbes on the C sample set. The accuracy of classification model was 0.857. The AUC was 0.88 (**Figure  8D**).

#### Classification model based on fecal metabolites and gut microbes on the C sample set

**Figure 9:**
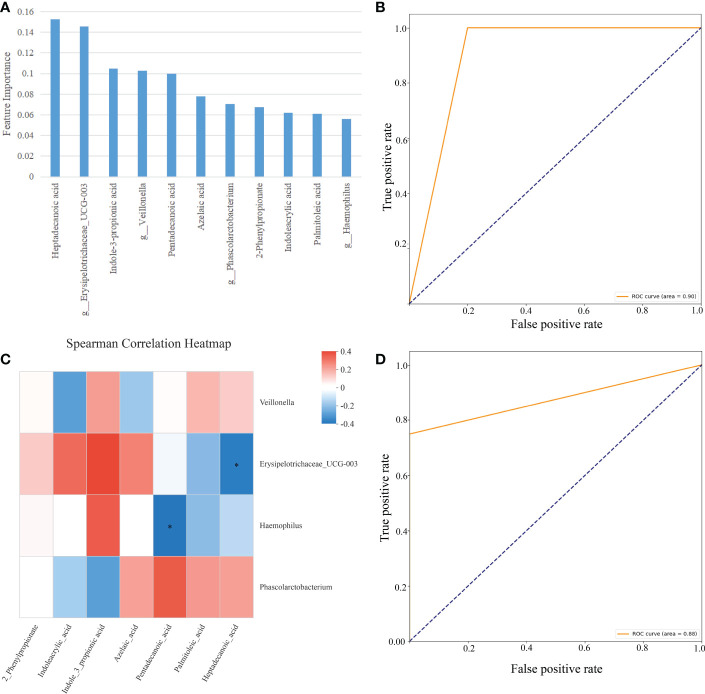
Classification model based on fecal metabolites and gut microbes on the C sample set. **(A)** The importance in descending order (7 fecal metabolites and 4 gut microbes). **(B)** Evaluation using ROC curve on the C sample set. **(C)** Spearman correlation heatmap, Spearman’s correlation between 7 fecal metabolites and 4 gut microbes, the abscissa is 7 metabolites and the ordinate is 4 intestinal flora, the color scale represents the spearman r value, with red and blue indicating positive and negative correlations, respectively, and *p< 0.05. **(D)** Evaluation using ROC curve on the C sample set (2 fecal metabolites and 2 gut microbes).

As shown in **Figure  9**, we used RF and SVM to calculate the features importance (7 fecal metabolites and 4 gut microbes) and trained a classification model on the C sample set. The importance of 7 fecal metabolites and 4 gut microbes in descending order was *Heptadecanoic acid* (0.152), *g:Erysipelotrichaceae_UCG-003*(0.146), *3-Indolepropionic acid*(0.104), *g:Veillonella*(0.102), *Pentadecanoic acid*(0.100), *Azelaic acid*(0.078), *g:Phascolarctobacterium*(0.070), *2-Phenylpropionate*(0.068), *Indoleacrylic acid*(0.061), *Palmitoleic acid*(0.061), *g:Haemophilus*(0.056)(**Figure  9A**). The accuracy of classification model was 0.857. And the AUC was 0.90 (**Figure  9B**). The results have shown that 7 fecal metabolites and 4 gut microbes could distinguish CAG patients from healthy controls.

Then we conducted the Spearman’ s rank correlation analyses to discovery the correlation between 7 metabolites and 4 gut microbiotas on the C sample set. Interestingly, Heptadecanoic acid was significantly negatively correlated to *Erysipelotrichaceae_UCG-003* (**Figure  9C**, R=-0.347, P=0.048, p < 0.05); Pentadecanoic acid was significantly negatively correlated to *Haemophilus* (**Figure  9C**, R=-0.364, P=0.037, P < 0.05);

The above result showed that 2 correlated fecal metabolites and 2 correlated gut microbes maybe more imported for CAG diagnosis. So, we constructed SVM classification model using 2 correlated fecal metabolites and 2 correlated gut microbes on the C sample set. The accuracy of classification model was 0.857. The AUC was 0.88 (**Figure  9D**).

## Discussion

Although gastroscopy with tissue biopsy is the most used technology for CAG diagnosis (Chooi et al., 2012; Rodriguez-Castro et al., 2018), it is always an uncomfortable experience for CAG patients because of its invasive process, fasting, eating laxatives, esophagus injury, nausea and vomiting as well as psychological pressure. Therefore, new non-invasive effective methods for CAG diagnosis in clinic is very urgent.

Disturbed metabolites in blood are often associated with different diseases (Yu et al., 2011; Xu et al., 2017; Zu et al., 2020; Coker et al., 2022; Wang et al., 2022). Previous researches indicated that in blood plasma, fifteen identified metabolites contributed most to the differentiating between CSG and GC, and characterized different stages of GC. 2-hydroxybutyrate, pyroglutamate, glutamate, asparagine, azelaic acid, ornithine, urate, 11-eicosenoic acid, 1-monohexadecanoylglycerol and g-tocopherol were increased, while creatinine, threonate were decreased in GC patients, indicating that oxidative stress and perturbed metabolism of amino acids and fatty acids might be invovled in the pathological process of GC (Yu et al., 2011). However, as to CAG, in CAG rats, 19 plasma metabolites and 18 urine metabolites were enrolled to construct the circulatory and excretory metabolome of CAG rats, which was in response to alterations of energy metabolism, inflammation, immune dysfunction, as well as oxidative stress. Seven plasma biomarkers and 7 urine biomarkers were screened to elucidate the pathogenesis of CAG based on the further correlation analysis with biochemical indexes. Finally, 3 plasma biomarkers (arginine, succinate and 3-hydroxybutyrate) and 2 urine biomarkers (α-ketoglutarate and valine) highlighted the potential to indicate risks of CAG in virtue of correlation with pepsin activity and ROC analysis (Cui et al., 2017). However, characteristic metabolites profiles of CAG in patients has not been well-clarified yet. Moreover, metabolites profiles, gut microbiota profiles as well as crosstalk between bacteria and metabolites in feces of CAG patients has not been clarified yet. In our present study, metabolites profiles, gut microbiota profiles as well as the possible crosstalk between bacteria and metabolites in feces of CAG patients were clarified, moreover, the biomarkers including metabolites and gut microbiota for CAG patients were also identified.

RF is a classifier containing multiple decision trees, each of trees is a classifier. For an input sample, N trees will have N classification results (Reynolds et al., 2019). RF integrates the results of all the classification votes, designating the category with the highest number of votes as the file output, which is equivalent to sampling both the sample and the features, thus enhancing generalization. The main advantages of the RF algorithm are: the small variance of the trained module, its generalization ability and its insensitivity to partially missing features due to the use of random sampling (Noble, 2006). Then ROC curves were used to evaluate the classification ability of the model.

In the present study, we demonstrated that there were 35 metabolites significantly changed in the feces of CAG patients in A sample set, compared with healthy volunteers. Using RF, 7 fecal metabolites (heptadecanoic acid, azelaic acid, indoleacrylic acid, indole-3-propionic acid, pentadecanoic acid, palmitoleic acid, 2-phenylpropionate) were selected from A sample set, to classify CAG from healthy people, as indicated by AUC on the A set. SVM is very powerful classifiers in complex datasets compared to the other many machine methods (Reynolds et al., 2019). It aims to create a decision boundary between two classes that enables the prediction of labels from one or more feature vectors (Noble, 2006). SVM as a classifier has been used in cancer classification (Reynolds et al., 2019; Huang et al., 2018) and biomarker selection (Zhang et al., 2021), since the high throughput microarray gene expression data was available in the early 2000’s. In our present study, after constructing SVM classification model using 7 fecal metabolites, the accuracy of classification model was 0.71, and the AUC was 0.71 on the C sample set. Therefore, metabolites disturbance indeed involves in the process of CAG, and could clarify CAG from healthy volunteers.

Gut microbiota lives in the gastrointestinal tract, and involving in modulating gastrointestinal function through producing functional molecules and metabolites, and interacting with the host metabolism (Morrison and Preston, 2016; Zhang et al., 2018). Healthy gut microbiota transplantation could recover inflammatory bowel diseases induced by gut microbiota disturbance (Tung et al., 2011; Li et al., 2017). Intestinal microbiota alteration in patients with CAG resulted in the reduced secretion of gastric acid and also contributed to the progression from IM to gastric cancer (Sharma et al., 1984; Park et al., 2019; Zhang et al., 2019; Zhou et al., 2021). However, the detailed relation of intestinal microbiota and CAG has been poorly investigated. In present study, the abundance of many fecal bacteria was significantly altered in CAG patients, compared with healthy volunteers. Then we used RF to select features of fecal bacteria for CAG patients. By using RF, 4 gut microbes (*g_Phascolarctobacterium*, *g_Erysipelotrichaceae_UCG-003*, *g_Veillonella*, *g_Haemophilus*) were selected as the features to classify CAG from healthy volunteers in B sample set. After constructing SVM classification model using 4 gut microbes, and the accuracy of classification model was 0.857 and the AUC was 0.88 on the C sample sets. Thus, fecal microbiota alteration especially *g_Phascolarctobacterium*, *g_Erysipelotrichaceae_UCG-003*, *g_Veillonella*, *g_Haemophilus*, could be as biomarkers for CAG patients.

There is a crosstalk between gut microbiota and metabolites (Wang and Zhao, 2018; Jia et al., 2021; Yang and Cong, 2021). However, up to nowadays, there is no research demonstrating the crosstalk between gut microbiota and metabolites in feces of CAG patients. In present study, RF and SVM were used to calculate the features importance (including the above 7 fecal metabolites and the above 4 gut microbes) and trained a classification model on the C sample sets. The accuracy of classification model was 0.857, and the AUC was 0.90. The results have shown that 7 fecal metabolites and 4 gut microbes could distinguish CAG patients from healthy volunteers. And it also indicated that it might be better to use features including fecal gut microbiota and fecal metabolites, than that of only using gut microbiota or metabolites to clarify CAG from healthy people, indicating there might be a crosstalk between fecal-derived microbiota and metabolites.

Therefore, we further used Spearman’s rank correlation analysis to predict the possible fecal-derived gut microbiota-metabolites crosstalk in CAG patients in the C sample set. Interestingly, in the selected above 7 fecal metabolites and the above 4 gut microbes, heptadecanoic acid was significantly negatively correlated to *Erysipelotrichaceae_UCG-003*; and pentadecanoic acid was significantly negatively correlated to *Haemophilus*, indicating a possible intricate relationship between fecal microbiota and fecal metabolites, such as heptadecanoic acid, *Erysipelotrichaceae_UCG-003*, pentadecanoic acid, *Haemophilus*. We further constructed SVM classification model using 2 correlated fecal metabolites and 2 correlated gut microbes on the C sample sets. The accuracy of classification model was 0.857, and the AUC was 0.88. The accuracy of classification model and AUC with heptadecanoic acid, *Erysipelotrichaceae_UCG-003*, pentadecanoic acid, *Haemophilus*, was similar with that with 4 gut microbiota and 7 metabolites, indicating there is possibly a crosstalk between heptadecanoic acid and *Erysipelotrichaceae_UCG-003*, as well as pentadecanoic acid and *Haemophilus* in the feces of CAG patients, and fecal-derived microbiome-metabolites crosstalk possibly involves in the pathological process of CAG, which should be further clarified and confirmed with a microbiome-based study based on shotgun metagenomics and metatranscriptomics. Therefore, the microbiota and the microbial-associated metabolites are possily potential diagnostic biomarkers and therapeutic targets for CAG.

Erysipelotrichi belongs to the Firmicutes phylum, and the bacterial family *Erysipelotrichaceae* which are immunogenic and possibly inter-host variation, and highly increased in mouse models of inflammatory bowel diseases (IBD) (Zhao et al., 2013; Palm et al., 2014; Dinh et al., 2015; Kaakoush, 2015), but significantly lowered in IBD patients (Dey et al., 2013; Gevers et al., 2014). Interestingly, and in the lumen of gastrointestinal tract of patients with colorectal cancer, the abundance level of *Erysipelotrichaceae* was significantly enriched (Chen et al., 2012; Zhu et al., 2014). *Erysipelotrichi* also appear to affect cholesterol and lipid metabolism in the GI tract (Parmentier-Decrucq et al., 2009). Distinct functional roles for the UCG-003 subtype have not been reported (Singh et al., 2019). The FUT2 loss-of-function mutations are very common and related with inflammatory bowel disease (IBD). Researchers further found that FUT2 loss-of-function mutations also increased CD8^+^ inducing *Alistipe* and *Phascolarctobacterium* and Th17 inducing *Erysipelotrichaceae UCG-003* in IBD patients (Cheng et al., 2021). In present study, interestingly, compared with healthy volunteers, the abundance of *Erysipelotrichaceae UCG-003* was significantly lowered in feces of CAG patients, indicating CAG might be a compensation condition against GC progress, and *Erysipelotrichaceae UCG-003* might be closely related with CAG. However, the detailed underlying molecular mechanism of *Erysipelotrichaceae UCG-003* on CAG still needed to be clarified.

Previous researches demonstrated that lipid metabolism involving in GC progress (Yu et al., 2011). Both pentadecanoic acid and heptadecanoic acid are multifaceted odd-chain fatty acids (OCFA) (Pfeuffer and Jaudszus, 2016), pentadecanoic acid and heptadecanoic acid can also be synthesized endogenously, for example, from gut-derived propionic acid (3:0) (Pfeuffer and Jaudszus, 2016), although most gut microbial propionic acid is absorbed and mostly metabolized by the liver (Al-Lahham et al., 2010). A number of studies have shown an inverse association between OCFA concentrations in human plasma phospholipids or RBCs and risk of type 2 diabetes and cardiovascular disease (Hodge et al., 2007; Patel et al., 2010; Mozaffarian et al., 2013; Santaren et al., 2014; Pfeuffer and Jaudszus, 2016). Heptadecanoic acid was proved to inhibit cell proliferation in PC-9 non-small-cell lung cancer cells with acquired gefitinib resistance *in vitro* (Xu et al., 2019). Therefore, the increased heptadecanoic acid in the feces of CAG patients might be associated with the decreased absorption into host or helping host to defeat against the pathological changes of stomach in CAG patients. However, up to nowadays, the relation of heptadecanoic acid and *Erysipelotrichaceae_UCG-003*, and pentadecanoic acid and *haemophilus*, has not been clarified yet. And we will further clarify the crosstalk between heptadecanoic acid and *Erysipelotrichaceae_UCG-003*, and pentadecanoic acid and *haemophilus*, and how to modulate the pathological process of CAG in the next study.

In conclusion, heptadecanoic acid, *Erysipelotrichaceae_UCG-003*, pentadecanoic acid, *haemophilus* were the potential biomarkers for CAG diagnosis in clinic. And heptadecanoic acid is the most potential biomarker for CAG diagnosis, and possibly involving in the pathological process of CAG. Furthermore, microbiome-metabolites crosstalk possibly involves in the pathological process of CAG, which should be further clarified and confirmed.

## Data availability statement

The datasets presented in this study can be found in online repositories. The names of the repository/repositories and accession number(s) can be found in the article/**Supplementary Material**.

## Ethics statement

The studies involving human participants were reviewed and approved by Medical Ethical Committee of Shuguang Hospital. The patients/participants provided their written informed consent to participate in this study. This study was approved by the Medical Ethical Committee of Shuguang Hospital (2020-834-41-01). All participants signed the informed consent.

## Author contributions

XG, PQ, and BG did most of experiments and wrote the original draft. YZ and ZF did data analysis, diagnostic marker acquisition and ROC evaluation. DY, CZ, YC, JN, collected fecal samples and diagnostic information from subjects. JL guided on collection of stool samples. DY and CZ took part in the fecal microbial and metabolic data analysis, respectively. JZ, HS, and GL designed experiments and wrote the manuscript. All authors contributed to the article and approved the submitted version.
