# Supplementary material for: Heptadecanoic acid and pentadecanoic acid crosstalk with fecal-derived gut microbiota are potential non-invasive biomarkers for chronic atrophic gastritis
Source: Front Cell Infect Microbiol. 2023 Jan 9;12:1064737. doi: 10.3389/fcimb.2022.1064737 (PMC9868245; doi:10.3389/fcimb.2022.1064737)
Supplement: Supplementary file 1 [file DataSheet_1.docx]

**Title Page**

**Heptadecanoic Acid and Pentadecanoic Acid Crosstalk with Fecal-derived Gut Microbiota are Potential Non-invasive Biomarkers for Chronic Atrophic Gastritis**

Xiao Gai^#,1^, Peng Qian^#,1^, Benqiong Guo^#,1^, Yixin Zheng^1^, Zhihao Fu^2^, Decai Yang^1^, Chunmei Zhu^1^, Yang Cao^1^, Jingbin Niu^1^, Jianghong Ling^3^, Jin Zhao^*,2^, Hailian Shi^*,4^, Guoping Liu^*,1^

Affiliations:

^1^School of Basic Medical Sciences, Shanghai University of Traditional Chinese Medicine, Shanghai 201203, China

^2^School of Computer Science, Fudan University, Shanghai 200438, China

^3^Department of Gastroenterology, Shuguang Hospital, Shanghai University of Traditional Chinese Medicine, Shanghai 201203, China

^4^Shanghai Key Laboratory of Compound Chinese Medicines, The Ministry of Education (MOE) Key Laboratory for Standardization of Chinese Medicines, The SATCM Key Laboratory for New Resources & Quality Evaluation of Chinese Medicine, Research Center of Shanghai Traditional Chinese Medicine Standardization, Institute of Chinese Materia Medica, Shanghai University of Traditional Chinese Medicine, Shanghai, 201203, China.

Correspondence to: Guoping Liu, E-mail address:13564133728@163.com；Phone: (+86) 21-5132-2286; Fax: (+86) 21-5132-2447. Hailian Shi, E-mail address: shihailian2003@163.com; Phone: (+86) 21-5132-2577; Fax: (+86) 21-5132-2505. Jin Zhao, E-mail address: jzhao@fudan.edu.cn; Phone: (+86) 21-3124-2255; Fax: (+86) 21-3124-2259; Corresponding address: School of Basic Medical Sciences (GP Liu), Institute of Chinese Materia Medica (HL Shi), Shanghai University of Traditional Chinese Medicine, 1200 Cailun Road, Zhangjiang Hi-tech Park, Shanghai 201203, China. School of Computer Science, Fudan University, Shanghai 200438, China (J Zhao).

^#^: These three authors contributed equally.

Running title: Non-invasive Biomarkers for CAG

**Table legends**

Table S1 Significant alteration of fecal metabolites of CAG_a group *vs* HC_a group.

The variation of fecal metabolites were indicated by arrows,“↑”or“↓”represents significant increase or decrease in CAG_a group, compared with HC_a group.

Table S2 Significant alteration of fecal metabolites of CAG_c group *vs* HC_c group.

The variation of fecal metabolites were indicated by arrows,“↑”or“↓”represents significant increase or decrease in CAG_c group, compared with HC_c group.

**Figure legends**

Figure S1 Pie chart of Class distribution of detected fecal metabolites

As shown in the pie chart, the value in parentheses after each metabolite category was the number of metabolites in that category, and the percentage of the number of metabolites in all identified metabolites in the results.

Table S1

| No. | Fecal metabolites | Class | P value | log2FC | Trend |
| --- | --- | --- | --- | --- | --- |
| 1 | Arginine | Amino Acids | 0.048 | 1.187475923 | ↑ |
| 2 | Sarcosine | Amino Acids | 0.044 | 0.520184622 | ↑ |
| 3 | Alanine | Amino Acids | 0.026 | 0.220712097 | ↑ |
| 4 | GABA | Amino Acids | 0.001 | 1.6413715 | ↑ |
| 5 | Asparagine | Amino Acids | 0.049 | 0.513733758 | ↑ |
| 6 | Valine | Amino Acids | 0.031 | 0.312855442 | ↑ |
| 7 | Phenylpyruvic acid | Benzenoids | 0.005 | -1.818732953 | ↓ |
| 8 | Gallic acid | Benzoic Acids | 0.013 | 0.961017612 | ↑ |
| 9 | 3-Aminosalicylic acid | Benzoic Acids | 0.006 | 2.08817979 | ↑ |
| 10 | LCA-3S | Bile Acids | 1.92813 E-04 | 1.298573165 | ↑ |
| 11 | apoCA | Bile Acids | 0.003 | 2.297934843 | ↑ |
| 12 | Gluconolactone | Carbohydrates | 0.041 | 0.615773753 | ↑ |
| 13 | Carnitine | Carnitines | 0.02 | 1.041473452 | ↑ |
| 14 | Azelaic acid | Fatty Acids | 3.65E-07 | -2.119852278 | ↓ |
| 15 | Myristic acid | Fatty Acids | 0.01 | 0.645041957 | ↑ |
| 16 | Pentadecanoic acid | Fatty Acids | 3.71E-05 | 1.300565309 | ↑ |
| 17 | Palmitoleic acid | Fatty Acids | 4.67E-06 | 1.330833274 | ↑ |
| 18 | Citramalic acid | Fatty Acids | 0.037 | 1.001010848 | ↑ |
| 19 | Heptadecanoic acid | Fatty Acids | 6.70E-07 | 2.466436779 | ↑ |
| 20 | Oleic acid | Fatty Acids | 0.027 | 0.919627732 | ↑ |
| 21 | Indoleacrylic acid | Indoles | 3.26E-05 | -2.542357645 | ↓ |
| 22 | Indole-3-propionic acid | Indoles | 4.08E-05 | -1.876473547 | ↓ |
| 23 | alpha-Ketoisovaleric acid | Organic Acids | 0.013 | -1.500853444 | ↓ |
| 24 | Ketoleucine | Organic Acids | 0.013 | -2.146771339 | ↓ |
| 25 | 3-Methyl-2-oxopentanoic acid | Organic Acids | 0.015 | -1.988467516 | ↓ |
| 26 | 4-Hydroxyphenylpyruvic acid | Phenols | 0.001 | -1.799826393 | ↓ |
| 27 | p-Hydroxyphenylacetic acid | Phenols | 0.026 | 1.976988618 | ↑ |
| 28 | 2-Phenylpropionate | Phenylpropanoic Acids | 9.30E-05 | -2.235458733 | ↓ |
| 29 | Hydrocinnamic acid | Phenylpropanoic Acids | 0.013 | -1.149756833 | ↑ |
| 30 | Phenyllactic acid | Phenylpropanoic Acids | 0.012 | 1.039430036 | ↓ |
| 31 | Cinnamic acid | Phenylpropanoids | 0.006 | -4.067011653 | ↓ |
| 32 | Ethylmethylacetic acid | SCFAs | 0.024 | 0.643508993 | ↑ |
| 33 | Propionic acid | SCFAs | 0.036 | 0.490298118 | ↑ |
| 34 | Isobutyric acid | SCFAs | 0.031 | 0.42167473 | ↑ |
| 35 | Isovaleric acid | SCFAs | 0.041 | 0.457851368 | ↑ |

Table S2

| NO. | Fecal metabolite | Class | P value | log2FC | Trend |
| --- | --- | --- | --- | --- | --- |
| 1 | Glutamic acid | Amino Acids | 0.028 | 0.840952152 | ↑ |
| 2 | Dimethylglycine | Amino Acids | 0.004 | 1.049317601 | ↑ |
| 3 | 1H-Indole-3-acetamide | Indoles | 0.015 | 1.801909049 | ↑ |
| 4 | Lactic acid | Organic Acids | 0.014 | 1.30814069 | ↑ |
| 5 | 2-Phenylglycine | Amino Acids | 0.017 | 2.656401091 | ↑ |
| 6 | Gallic acid | Benzoic Acids | 0.008 | 1.717631639 | ↑ |
| 7 | 3,4-Dihydroxyhydrocinnamic acid | Phenylpropanoids | 0.006 | 3.108058375 | ↑ |
| 8 | Hydroxyphenyllactic acid | Phenylpropanoic Acids | 0.037 | 0.711097075 | ↑ |
| 9 | Aspartic acid | Amino Acids | 0.011 | -1.191536604 | ↓ |
| 10 | Gluconolactone | Carbohydrates | 0.014 | 1.92634957 | ↑ |
| 11 | Pyroglutamic acid | Amino Acids | 0.011 | -1.053719756 | ↓ |
| 12 | 3-Hydroxybutyric acid | Organic Acids | 0.019 | 1.666296736 | ↑ |
| 13 | 2-Hydroxybutyric acid | Organic Acids | 0.012 | 1.37956068 | ↑ |
| 14 | Propionic acid | SCFAs | 0.045 | 0.724252631 | ↑ |
| 15 | Homovanillic acid | Phenols | 0.028 | 2.174524955 | ↑ |
| 16 | 3-Hydroxyphenylhydracrylic acid | Phenylpropanoic Acids | 0.010 | 3.642572665 | ↑ |
| 17 | Mandelic acid | Benzenoids | 0.001 | 0.348820211 | ↑ |
| 18 | Oxalic acid | Organic Acids | 0.012 | 1.053069553 | ↑ |
| 19 | Phthalic acid | Benzoic Acids | 0.001 | 1.241821054 | ↑ |
| 20 | 7-DHCA | Bile Acids | 0.007 | 5.045953213 | ↑ |
| 21 | 3-DHCA | Bile Acids | 0.037 | 1.980345563 | ↑ |
| 22 | Murocholic acid | Steroids and steroid derivatives | 0.009 | 4.05648179 | ↑ |
| 23 | bHDCA | Bile Acids | 0.017 | 4.379016112 | ↑ |
| 24 | Pentadecanoic acid | Fatty Acids | 0.014 | 1.141644157 | ↑ |
| 25 | Dihomo-gamma-linolenic acid | Fatty Acids | 0.012 | 2.959930391 | ↑ |
| 26 | DPA | Fatty Acids | 0.015 | 2.98016112 | ↑ |
| 27 | Adrenic acid | Fatty Acids | 0.014 | 1.253208872 | ↑ |
| 28 | Heptadecanoic acid | Fatty Acids | 0.001 | 2.006438007 | ↑ |
| 29 | 7-KetoLCA | Bile Acids | 0.014 | 4.406382299 | ↑ |

**Figure S1**

**
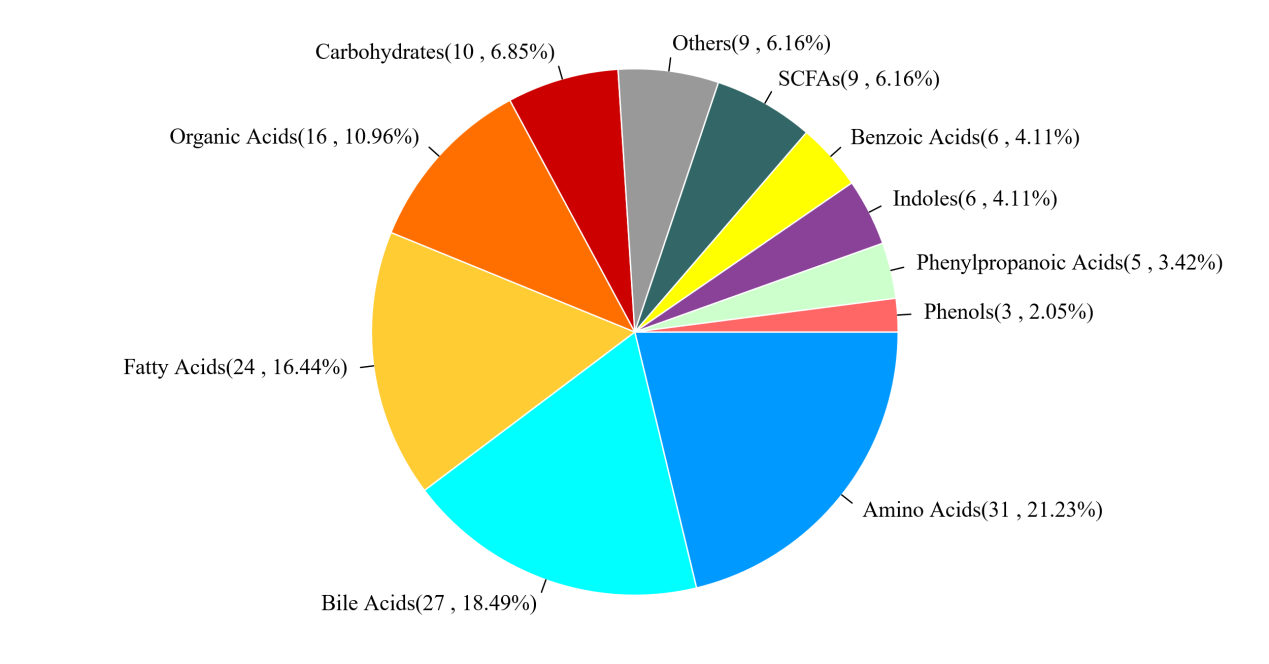
**
